# The Influence of Work–Family Conflict on Burnout during the COVID-19 Pandemic: The Effect of Teleworking Overload

**DOI:** 10.3390/ijerph181910302

**Published:** 2021-09-30

**Authors:** Holger Raúl Barriga Medina, Ronald Campoverde Aguirre, David Coello-Montecel, Paola Ochoa Pacheco, Milton Ismael Paredes-Aguirre

**Affiliations:** 1 Facultad de Ciencias Sociales y Humanísticas, Escuela Superior Politécnica del Litoral (ESPOL), Campus Gustavo Galindo, Km. 30.5 Vía Perimetral, Guayaquil 090902, Ecuador; recampov@espol.edu.ec; 2 ESPAE Graduate School of Management, Escuela Superior Politécnica del Litoral (ESPOL), Campus Las Peñas, Malecón No. 100 y Loja, Guayaquil 090306, Ecuador; dacoello@espol.edu.ec; 3 EGADE Business School, Tecnológico de Monterrey, Carlos Lazo 100, Santa Fé 01389, Mexico

**Keywords:** work–family conflict, family–work conflict, burnout, teleworking, work overload, COVID-19

## Abstract

If there is any field that has experienced changes as a result of the COVID-19 pandemic, it is work, primarily due to the implementation of teleworking and the effort made by workers and families to face new responsibilities. In this context, the study aims to analyze the impact of work–family conflict on burnout, considering work overload, in teleworkers during the COVID-19 pandemic. To evaluate the hypotheses, we used data collected during the last week of July 2020 using an online survey. Work–family conflict and burnout were measured using the Gutek et al. (1991) and Shirom (1989) scales. We tested the hypotheses using a structural equation model (SEM). The results indicated, between other findings, that there was a positive relationship between work–family conflict and family–work conflict and all the dimensions of burnout. However, there was no effect of teleworking overload in the work–family conflict and burnout relationship. This article is innovative because it highlights the importance of the economic and regulatory conditions that have surrounded the modality of teleworking during the pandemic, and their influence on wellbeing and psychosocial risks in workers.

## 1. Introduction

The COVID-19 pandemic brought significant changes, not only in the field of public health with the adoption of strict confinement and biosecurity measures, but also in the field of work and employment. The adoption of teleworking worldwide as the first option to continue operations and provide services, especially during the second quarter of 2020, was one of the most remarkable transformations regarding workplaces and working conditions [1,2]. Although teleworking as a working modality was not new, its adoption as a response to contain the spread of the pandemic enhanced the relevance of several challenges that must be addressed, not only by academics but by employers and policymakers. Among these challenges are the work organization, safety and occupational health, the rapid acquisition of competencies (e.g., digital skills), and the simultaneous coexistence of the work and family spheres in the same physical space [3,4,5].

According to the International Labour Organization (ILO) [6], before the COVID-19 pandemic, 7.9% of the global workforce (approximately 260 million workers) had adopted teleworking as a working modality. However, the mobility restrictions derived from the pandemic significantly increased these numbers, especially in occupations that could be carried out with limited physical contact. Despite the limitations in measuring working from home during the pandemic, the ILO [6] estimated that during the second quarter of 2020, 17.4% of the world’s workers worked from home, this proportion being higher in high-income countries (25.4%) than in upper–middle- (17.1%) or lower–middle- and low-income countries (13.6%).

The teleworking panorama in Latin America and the Caribbean was heterogeneous due to, among other factors, the access to the technologies necessary for this working modality and the socioeconomic differences between countries. During the second quarter of 2020, the proportion of workers working from home was higher in Argentina (22.2%), Costa Rica (19.8%), Chile (19.1%), Peru (16.3%), and Mexico (15.1%). In contrast, Brazil and Ecuador reported lower proportions of workers who adopted teleworking; 10.7% and 10.6%, respectively. Due to the region’s disparities, employees with higher education and who worked in professional, technical, administrative, or managerial occupations adopted teleworking in a greater proportion than informal or low-skilled young workers [6].

Due to the COVID-19 pandemic, the health emergency was declared in Ecuador on 16 March 2020 by Decree 1017 [7]. During March and the first days of April 2020, the healthcare system in Guayaquil (Ecuador) exceeded its capacity. According to the Ecuadorian Civil Registry Office [8], the death toll in Guayaquil exceeded 400 people per week from March to May. As a result of the health emergency that the country, especially Guayaquil, was going through due to the rapid increase of COVID-19 positive cases, general quarantine and strict confinement measures were established.

According to the Ecuadorian Ministry of Labour statistics [9,10], a total of 14 thousand workers had adopted teleworking as a working modality from its introduction in 2016 [11] until before the health emergency. At the beginning of the pandemic, more than 208 thousand workers adopted teleworking as their primary working modality [9], while at the end of the first quarter of 2020, the number of teleworkers increased by 56% (325 thousand workers), and by the end of July 2020, more than 433 thousand workers adopted this modality. Currently, a little more than 457 thousand workers [10], representing approximately 6% of employed workers, continue to telework.

Before the pandemic, the Ecuadorian Ministry of Labour issued Ministerial Agreement No. MDT-2016-190 [11], which regulated teleworking in the private sector. Nevertheless, during the pandemic, several regulations regarding teleworking were developed by the Ministry of Labour [12,13] or approved by the National Assembly [14]. For example, the Ministerial Agreement No. MDT-2020-076 [12] established a general framework for applying teleworking during the health emergency declaration. In order to promote economic and productive reactivation, as well as the maintenance of employment conditions, the Organic Humanitarian Act [14] (approved on 19 June 2020) established that employers might reduce the working day by up to 50% for up to one year from the entry into force of the law, among other factors concerning to teleworking. In addition, following the provisions of the Organic Humanitarian Act, the Ministerial Agreement No. MDT-2020-181 [13] extended the current regulation regarding workers’ rights under this working modality, the establishment of the right to disconnection, and the registration of workers under this modality.

### 1.1. Work–Family Conflict

Work–family conflict has been widely discussed in scholarly literature [15,16,17,18,19,20]; nevertheless, its relevance has increased during the last years [21]. Netemeyer et al. [22] conceived it as a two-way psychological phenomenon that includes the work–family conflict and family–work conflict. From this theoretical conception, work–family conflict can also be considered a type of role conflict [23]. A role conflict is the simultaneous presence of two or more sets of pressures so that the fulfilment of one role (e.g., professional role) makes difficult the fulfilment of the others (e.g., family role) [24].

Even though an incompatibility might exist between work and family roles, it does not mean that they cannot support each other. Kahn et al. [25] mentioned a conflict between both roles because of their opposing characteristics. Hence, work–family conflict can be defined as an inter-role conflict that has three approaches: time-based conflict, strain-based conflict, and behavior-based conflict. Amstad et al. [26] pointed out that work–family conflict occurs when experiences and commitments at work interfere with family life. In contrast, family–work conflict arises when family responsibilities interfere with work obligations. Cifre and Salanova [27] established that family–work conflict influences a deterioration in parental function and enhances adverse outcomes such as irritation, anxiety, and depression, among others. For their part, Palmer et al. [28] mentioned that work–family conflict is associated with negative results such as physical tension, burnout, job dissatisfaction, poor job performance, and intention to leave work. Mansour and Tremblay [29] found that psychosocial safety climate is negatively related to work–family conflict, and family-supportive supervisor behavior mediates this relationship.

According to the role conflict theory, work and family-related factors can boost role conflict [30]. The role conflict perspective is based on two premises. The first premise establishes that the demands derived from performing multiple simultaneous roles (e.g., work and family roles) lead to the experience of role conflict [31] because individuals have limited time and energy. The second premise indicates that the experience of role conflict causes psychological distress and burnout, which reduce the sense of wellbeing [32].

Additionally, according to Bakker and Demerouti [33], the job demands–resources (JDR) model suggests that job demands are precursors of a health damage process. Job resources are advocates of a motivational process. The JDR model specifies how demands and resources interact and predicts important organizational outcomes. Based on this approach, excessive job demands negatively affect work and family wellbeing, resulting in frustration and dissatisfaction [34]. Previous studies have shown that the JDR model can predict burnout when job demands are higher than job resources, and work engagement when job resources are higher than job demands [35,36].

### 1.2. Burnout

Burnout is possibly one of the most studied topics in the field of occupational health and psychosocial risks. There is extensive literature [37,38,39] that has addressed various aspects, such as those shown by the subsequent meta-analyses: the higher prevalence of burnout syndrome among surgical/urgency residencies than in clinical specialties [40]; the association between physician burnout and patient safety [41]; the relationship between burnout, depression, and anxiety [42], among others.

Moreover, numerous studies have analyzed several factors associated with burnout in recent years [43,44,45,46,47,48,49]. Guidetti et al. [43] proposed a theoretical framework to explain different types of burnout, taking into account the sense of guilt as a symptom. Ochoa [45] determined that the legal framework influences the burnout and organizational outcomes relation. For their part, Fiorilli et al. [47] and Duan et al. [46] determined that social support from different sources (e.g., family or peers) helps to reduce burnout. Duan et al. [46] highlighted that workplace violence promotes burnout syndrome among Chinese physicians. Based on the evidence from hospitality professionals, Koo et al. [48] identified that material rewards such as promotion and incentives reduce the perception of burnout. West et al. [50], in a systematic review and meta-analysis, determined that individual, structural, or organizational strategies can clinically reduce burnout among physicians. In addition, Mansour and Tremblay [51] explored that burnout mediates the relationship between psychosocial safety climate and safety workarounds. Their results concluded that burnout is negatively affected by psychosocial safety climate, and safety workarounds are positively influenced by burnout.

More recent research during the COVID-19 pandemic highlights the importance of the study of burnout [52,53,54], since this pandemic brought with it adverse psychosocial effects such as stress, fear, and frustrations [55], without leaving aside the impact that the confinement and the pandemic itself has had on workplaces and working conditions [56,57]. In this line, Yıldırım and Solmaz [58] explored the relationship between COVID-19 stress and COVID-19 burnout mediated by resilience, determining a positive relationship between stress and burnout. Similar results regarding the association between stress, anxiety, depression, and burnout were found by Luceño-Montero et al. [52]. Another study that highlights the factors that influence burnout in the context of the COVID-19 pandemic is the one carried out by Giusti et al. [59], where, based on a sample of health professionals, it was confirmed that the fear of infection enhances the sense of emotional exhaustion and depersonalization. At the same time, friend support reduces burnout in all its dimensions.

Regarding the relationship between work–family conflict and burnout, extensive literature analyzes this relationship in different contexts [37,60,61,62,63,64,65,66,67,68,69], even in the COVID-19 pandemic [70]. Moreover, several factors, such as coping strategies [71], organizational support [63], or psychological capital [72], have been evaluated as mediating or moderating variables. Likewise, the literature that addresses the issue of work overload and burnout is extensive [73,74]. This article analyzes the relationship between work–family conflict and burnout, incorporating the effect of teleworking overload during the COVID-19 pandemic.

There are several approaches to conceptualize and measure burnout, among which stand out the Maslach Burnout Inventory (MBI) [75], the Questionnaire for the Evaluation of the Burnout Syndrome at Work (CESQT, by its acronym in Spanish) [76,77], and the Shirom Burnout Scale [78]. In this study, burnout is considered as a response to chronic job stress that constitutes negative attitudes and feelings and is primarily characterized by the experience of physical fatigue, cognitive weariness, and emotional exhaustion. The first dimension Shirom [78] refers to is “physical fatigue” and refers to feelings of tiredness and low energy levels in carrying out daily tasks at work, such as getting up in the morning to go to work. The second dimension, “emotional exhaustion”, refers to feeling too weak to display empathy to clients and coworkers and lacking the energy needed to invest in relationships with other people at work. Finally, the third dimension, “cognitive weariness”, refers to feelings of slow thinking and reduced thinking agility. Each dimension of burnout covers the draining and depletion of energetic resources in a particular domain.

### 1.3. Teleworking

Telework is a polysemic term on which there is abundant literature [79,80,81,82]. It has evolved since its initial use by Nilles [83] in the 1970s, with the practice of working at home to avoid gasoline consumption in the United States due to the oil crisis of those years [83,84].

In a recent report dedicated to teleworking and its modalities, the ILO [85] has established that four concepts must be taken into account in the world of teleworking, such as “remote work”, “telework”, “work at home”, and “home-based work”. “Remote work” comprises situations where the work is fully or partly carried out on an alternative worksite other than the default place of work; it can also be performed in various possible locations. “Teleworking”, according to the ILO definition, is a subcategory of the broader concept of remote work. It includes workers who use information and communications technology (ICT) or landline telephones to work remotely. For its part, “work at home” refers to work that takes place fully or partly within the worker’s residence. Finally, the ILO points out that “home-based work: is a subcategory of “work at home”, and comprises the work carried at home, regardless of whether the worker’s home could be considered the default place of work.

There are various definitions of teleworking, with different approaches, of which we comment on some, by way of illustration of the variety and breadth of the concept. Garrett and Danziger [86] defined teleworking as “the work performed by (a) those whose remote work is from the home or a satellite office, (b) those whose telework is primarily in the field, and (c) those whose work is ‘networked’ in such a way that they regularly work in a combination of home, work, and field contexts”. Other definitions, such as those proposed by Fonner and Roloff [87] and Konradt et al. [88], emphasize technology’s partiality and contribution to this working modality. Konradt et al. [88] defined teleworking as a form of work organization in which the work is partially or entirely done outside the conventional company workplace with information and telecommunication services.

In Ecuador, according to the Organic Humanitarian Act [14], teleworking “consists of the performance of activities or the provision of services using the information and communication technologies as a support for the contact between the worker and the employer, without requiring his physical presence in a specific workplace (…). The workers under this modality will enjoy all individual and collective rights, as well as social benefits”. In addition, since its approval, both the Labour Code and the Organic Public Service Act (LOSEP, by its acronym in Spanish) recognize four types of teleworkers (autonomous, mobile, partial, and occasional).

During the COVID-19 pandemic, some studies have been published on teleworking [2,89,90]. Belzunegui-Eraso and Erro-Garcés [89] analyzed the implementation of teleworking on a massive scale during the first months of the pandemic. They highlighted the main problems in implementing this working modality, such as the impossibility of facilitating and installing equipment for workers to work remotely, among others. Andrade and Petiz Lousã [90] explored the relationship between teleworking and work–family conflict moderated by supervisor and coworker support in a Portuguese multi-professional sample. For their part, Pulido-Martos et al. [2] found that working modality moderates the relationship between social support and vigor at work. According to their results, hybrid work modality and teleworking reduce the influence of social support on vigor at work, since teleworking could decline the quality of professional relations.

In this study, we agree with the definition of teleworking proposed by Bailey and Kurland [84], who state that telework, also known as virtual work, involves the use of computer technology to work from home or another place outside the traditional office during a part of the week. In addition, we agree with the ILO [85] definition of teleworking, but with the nuance that the modality was not voluntary but required by biosafety measures during the pandemic.

### 1.4. Teleworking Overload Effects on Work–Family Conflict and Burnout

The ILO [3] mentions that the appearance of teleworking as a more recurrent work modality during the last years generates greater attention towards the study of the relationship between work–family conflict and teleworking. Struggles in work and family spheres management occur almost daily and have consequences for professional activities and personal life [91]. The usual approach of studying the relationship between these two variables is to explore the nature of interactions between work and family activities [92]. However, researchers highlight the importance of examining how this relationship varies when teleworking is applied outside regular office hours [93,94], when there is a change in its frequency [95], or when dedication is considered [96,97].

Other studies have focused on studying work–family conflict and teleworking [98], considering the context and organizational support [99], the working conditions [100], and the personal and family characteristics of the teleworker when doing their activities [100,101,102,103], among other factors. Different results arose, depending on the particularities and the context of the analysis. Despite all those previous findings, the work environment that brought the COVID-19 pandemic generates great interest in exploring the relationship between these variables, especially when teleworking increased family–work conflict work–family conflict at the same time.

Work overload is the perception of having too much to do [104,105] and can impact negatively on productivity [106], reduce organizational commitment, and increase absenteeism due to illness [107]. Various studies [106,108,109,110,111,112,113,114,115,116] have associated work overload with high levels of work–family conflict [117]. People who are often working from home change their working hours and experience an increased likelihood of interruptions and distractions because of the presence of their family, especially during the COVID-19 pandemic [118].

The teleworking context during the COVID-19 pandemic gives the impression that employees save commutation time; therefore, that saved time could be used to extend the working hours [119]. There is also the point of view that working hours can be easily extended given that the employee is at home. For this reason, employees have experienced an increase in the working hours and the job demands [2,120,121]. Considering that work and family responsibilities increased during the confinement due to the COVID-19 pandemic [3], it would be assumed that teleworking overload affects the relationship between work–family conflict and burnout.

As mentioned before, extensive literature addresses the relationship between work–family conflict and burnout [60,61,62,63,64,65,66,67,68,69,71]. Nevertheless, when this relationship is tested in the context of teleworking modality, the literature is diverse. It gives us broad concerns that were magnified by the COVID-19 pandemic, such as the work intensification [122], the increase of job-related worries [123], and the promotion of role ambiguity [124], among others. The existing literature raises the advantages of teleworking [125,126], but several negative impacts on workers’ health should be considered [62,124,125].

### 1.5. Research Objective and Hypotheses

**Figure 1:**
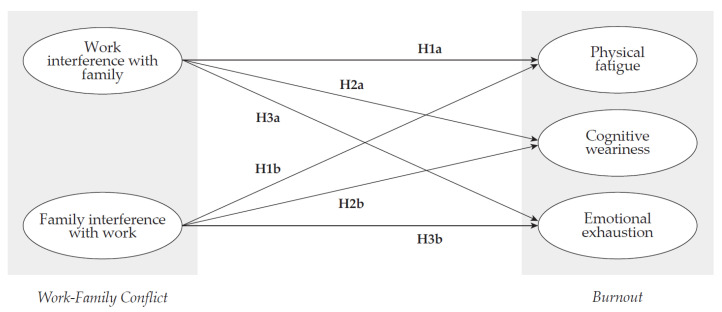
Research model.

Based on the literature mentioned above, this study tested the research model shown in Figure 1.

The objective of this article was to study the impact of work–family conflict on burnout, considering work overload, in teleworkers during the COVID-19 pandemic. Hence, the following research hypotheses were formulated:

**Hypothesis** **1a** **(H1a).** *Work interference with family is positively related to the physical fatigue dimension of burnout during the COVID-19 pandemic.*

**Hypothesis** **1b** **(H1b).** *Family interference with work is positively related to the physical fatigue dimension of burnout during the COVID-19 pandemic.*

**Hypothesis** **2a** **(H2a).** *Work interference with family is positively related to the cognitive weariness dimension of burnout during the COVID-19 pandemic.*

**Hypothesis** **2b** **(H2b).** *Family interference with work is positively related to the cognitive weariness dimension of burnout during the COVID-19 pandemic.*

**Hypothesis** **3a** **(H3a).** *Work interference with family is positively related to the emotional exhaustion dimension of burnout during the COVID-19 pandemic.*

**Hypothesis** **3b** **(H3b).** *Family interference with work is positively related to the emotional exhaustion dimension of burnout during the COVID-19 pandemic.*

**Hypothesis** **4** **(H4).** *The presence of teleworking overload affects the relationships between work–family conflict and burnout dimensions during the COVID-19 pandemic.*

To evaluate the hypotheses, we used data collected during the last week of July 2020 using an online survey. Work–family conflict and burnout were measured using the Gutek et al. [127] and Shirom [78] scales. We first conducted confirmatory factor analysis (CFA) using the maximum likelihood estimation method to validate the dimensionality of the different constructs included in this study. After that, we tested the hypotheses using a structural equation model (SEM). Both dimensions of work–family conflict (work interference with family and family interference with work) were considered as first-order independent variables. The three dimensions of burnout (physical fatigue, cognitive weariness, and emotional exhaustion) were the dependent variables. As in the CFA, we estimated SEM specifications using the maximum likelihood estimation method. We tested the effect of teleworking overload using a multigroup analysis.

This study is innovative for several reasons. First, the research reveals the psychosocial consequences of a new modality of work overload and teleworking during the COVID-19 pandemic. Second, this article is one of the few in Latin America that addresses work–family conflict and burnout and the influence of teleworking overload during the pandemic. Finally, the study highlights the importance of the economic and regulatory conditions and contingencies that have surrounded the modality of teleworking during the pandemic in Ecuador, which may have impacted consequences such as the normalization of a factor of high occupational risk, e.g., as work overload.

The article is organized as follows. Section 2 describes the materials and methods, discussing how the data was collected and analyzed. Results and a discussion are presented in Section 3 and Section 4, respectively. Finally, Section 5 highlights the conclusions along with the research limitations and perspectives.

## 2. Materials and Methods

### 2.1. Participants and Data Collection Procedure

The study design was cross-sectional, and a convenience sample was used. Data was collected during the last week of July 2020. At the time of the data collection, a return-to-work plan due to the COVID-19 pandemic had started in Guayaquil, which allowed workers to adopt total or partial teleworking modalities. Participants were recruited mainly through word-of-mouth and social networks. They received an invitation with an anonymous link allowing them to fill in an online survey. Participation was voluntary. All participants were asked to indicate that they agreed to participate in the study with an online informed consent form. In addition, participants were informed about the importance and objectives of the research and its confidentiality nature. We obtained 1240 responses from workers located in Guayaquil, Ecuador. After dropping those who did not fully answer the questionnaire, a total of 1044 valid surveys were considered.

Out of the 1044 respondents, 45% were male, and 55% were female. Regarding marital status, 60.6% of the participants were single, while 27.1% were married, 6.2% were in free union, 5% were divorced, and 1.1% were widowed. Most of the surveyed workers were millennials (60.2%) and had completed an academic degree (66.1%). The average work experience of the participants was ten years (SD = 20.42). Concerning the job positions of the respondents, 71.8% worked in operational roles, while 28.2% worked in supervisory roles. In all, 72.8% of the participants worked in private enterprises and 27.2% in public institutions. Regarding the industry where participants work, 65.3% indicated they worked in the services industry (44.3%) and the commerce industry (21%). In comparison, 34.7% indicated they worked in other industries (e.g., educational services, manufacturing, and agriculture, among others). Concerning teleworking, 30.8% of the respondents worked in this modality more than 8 h a day, while 69.2% indicated they teleworked 8 h or less a day.

### 2.2. Measures

Work–family conflict: This construct was measured using the eight-item scale developed by Gutek et al. [127], which defines that work–family conflict is composed of two dimensions: (i) work interference with family, and (ii) family interference with work. The items were scored on a five-point Likert scale ranging from “strongly disagree” (1) to “strongly agree” (5). Example items were, for work interference with family dimension, “After work, I come home too tired to do some of the things I’d like to do”, and for family interference with work dimension, “I’m often too tired at work because of the things I have to do at home”.

Burnout: Shirom’s [78] 14-item scale was used to measure burnout. This scale comprises three dimensions: (i) physical fatigue, (ii) cognitive weariness, and (iii) emotional exhaustion. The items were scored on a five-point Likert scale ranging from “strongly disagree” (1) to “strongly agree” (5). Example items were, for physical fatigue dimension, “I feel tired”, for cognitive weariness dimension, “My thinking process is slow”, and for emotional exhaustion dimension, “I feel I am unable to be sensitive to the needs of coworkers and customers”.

Teleworking overload: A single item was used to measure teleworking overload: “How many hours do you telework a day?”.

### 2.3. Data Analysis

To validate the dimensionality of the constructs included in this study, we conducted a confirmatory factor analysis (CFA) using the maximum likelihood estimation method. First, we conducted a CFA for the work–family conflict and burnout constructs. The CFA specifications were tested using the overall sample data as well as two subsamples. The first group (G_1_; overload = 0; *n_G1_* = 722) reported that they teleworked eight or fewer hours, while the second group (G_2_; overload = 1; *n_G2_* = 322) teleworked more than eight hours. Second, we estimated a measurement model to verify if all constructs were correlated with each other [128]. We removed items with factor loadings of <0.5 from the CFA specifications to ensure adequate convergent validity levels [129,130]. Given that the chi-square to degrees of freedom ratio (χ^2^/df) is sensitive to sample size, the comparative fit index (CFI), the goodness-of-fit index (GFI), the normed fit index (NFI), the Tucker–Lewis index (TLI), the root mean square error of approximation (RMSEA), and the standardized root mean square residual (SRMR) were used to evaluate the goodness of fit of the specifications to the data. Internal consistency of the dimensions of both constructs was assessed using the McDonald’s omega (ω) composite reliability coefficient rather than the Cronbach’s alpha (α) coefficient in order not to underestimate reliability when there is considerable variation in factor loadings [130,131,132,133,134].

Once we evaluated the construct validity, we tested the hypotheses using a structural equation model (SEM) where both dimensions of work–family conflict (work interference with family and family interference with work) were considered independent variables. The three dimensions of burnout (physical fatigue, cognitive weariness, and emotional exhaustion) were the dependent variables. As in the CFA, we estimated SEM specifications using the maximum likelihood estimation method considering specific analysis for the overall sample and subsamples (G_1_ and G_2_).

In this study, we tested the effect of teleworking overload using a multigroup analysis. Based on this, the equivalence of the hypothesized causal structure was evaluated using progressively restrictive nested models [135,136,137]. If the equivalence is demonstrated, then the effect of teleworking overload will be confirmed. Specifically, we estimated six nested specifications (M_1_, M_2_, …, M_6_). First, we estimated a baseline model for both groups (M_1_). This specification allowed measurement and structural loadings, intercepts, and residuals to be estimated freely. Afterwards, we incorporated constraints to the different parameters to be estimated to test the equivalence across both groups (G_1_ and G_2_). We assessed the goodness-of-fit of each of the specifications using a variety of indices (e.g., TLI, CFI, and RMSEA) and following the cutoffs suggested by Chen [138] (ΔRMSEA ≤ 0.02; ΔCFI ≤ 0.01). All models were estimated using IBM AMOS Version 24.0 for Windows.

## 3. Results

### 3.1. Descriptive Analysis

Table 1 shows the mean and standard deviation of the dimensions of burnout (physical fatigue, cognitive weariness, and emotional exhaustion) and work–family conflict (work interference with family and family interference with work). We computed factor scores following non-refined methods [139]. Each indicator that comprises the dimension was multiplied by its corresponding factor loading, then we summed the resulting scores and divided them by the sum of the factor loadings. This computation yielded weighted mean factor scores where higher scores represent higher burnout and work–family conflict, respectively. Regarding the overall sample, burnout dimensions showed medium scores in physical fatigue (2.39, SD = 0.90), cognitive weariness (2.19, SD = 0.91), and emotional exhaustion (2.23, SD = 0.91). The work–family conflict dimensions also showed medium scores. The participants indicated that the work interference with family is higher (2.85, SD = 0.95) than the family interference with work (2.34, SD = 0.89).

When comparing these dimensions between G_1_ (overload = 0) and G_2_ (overload = 1), some differences arose. Regarding burnout dimensions, physical fatigue scores were higher in G_2_ (2.45, SD = 0.93) than in G_1_ (2.37, SD = 0.89), cognitive weariness scores were similar in G_1_ (2.19, SD = 0.90) and G_2_ (2.18, SD = 0.93), and emotional exhaustion scores were higher in G_1_ (2.26, SD = 0.90) than in G_2_ (2.17, SD = 0.93). We did not find statistically significant differences in these three dimensions among both groups. Regarding work–family conflict dimensions, work interference with family scores were higher in G_2_ (3.13, SD = 1.02) than in G_1_ (2.73, SD = 0.89), and family interference with work scores were higher in G_1_ (2.35, SD = 0.87) than in G_2_ (2.31, SD = 0.94). A statistically significant difference was determined in work interference with family scores between G_1_ and G_2_ (*t* = 6.48, *p* < 0.01).

### 3.2. Confirmatory Factor Analysis and Reliability

We conducted various CFAs to validate the dimensionality of the constructs included in this study. Burnout was evaluated using a three-intercorrelated-factor structure (physical fatigue, cognitive weariness, and emotional exhaustion). At the same time, work–family conflict was assessed using a two-intercorrelated-factor specification (work interference with family and family interference with work).

According to the results for the overall sample, the goodness-of-fit indices for the burnout scale (CFI = 0.982; GFI = 0.963; NFI = 0.978; TLI = 0.976; RMSEA = 0.059 (90% CI: 0.052–0.066); SRMR = 0.028) as well as for the work–family conflict scale (CFI = 0.981; GFI = 0.977; NFI = 0.977; TLI = 0.969; RMSEA = 0.073 (90% CI: 0.058–0.088); SRMR = 0.038) were adequate. For the G_1_ subsample, the goodness-of-fit indices were appropriate for the burnout scale (CFI = 0.983; GFI = 0.961; NFI = 0.976; TLI = 0.977; RMSEA = 0.058 (90% CI: 0.049–0.067); SRMR = 0.026) and for the work–family conflict scale (CFI = 0.979; GFI = 0.975; NFI = 0.974; TLI = 0.966; RMSEA = 0.073 (90% CI: 0.056–0.092); SRMR = 0.037). In addition, fit indices were satisfactory for the G_2_ subsample when evaluating burnout (CFI = 0.965; GFI = 0.919; NFI = 0.951; TLI = 0.952; RMSEA = 0.086 (90% CI: 0.072–0.099); SRMR = 0.046) and work–family conflict (CFI = 0.982; GFI = 0.969; NFI = 0.972; TLI = 0.970; RMSEA = 0.075 (90% CI: 0.046–0.104); SRMR = 0.043) scales factorial structure. As shown in Table 2, all factor loadings were statistically significant (*p* < 0.01) and over the cutoff value of 0.5 for both constructs considering the overall sample and G_1_ and G_2_ subsamples, which ensured adequate levels of internal consistency [130].

McDonald’s omega (ω) coefficient estimates for evaluating reliability were satisfactory for each dimension of burnout and work–family conflict (see Table 3). For the burnout scale, ω values ranged from 0.882 (physical fatigue) to 0.945 (cognitive weariness) for the overall sample, from 0.885 (physical fatigue) to 0.943 (cognitive weariness) for the G_1_ subsample, and from 0.880 (physical fatigue) to 0.964 (cognitive weariness) for the G_2_ subsample. Concerning the work–family conflict scale, ω estimates for the work interference with family and family interference with work dimensions for the overall sample were 0.861 and 0.839, respectively; 0.835 and 0.830 for G_1_; and 0.893 and 0.859 for G_2_.

Once we validated the factorial structure of burnout and work–family conflict, we estimated a measurement model to identify if the dimensions of both constructs were intercorrelated. The goodness-of-fit indices of the five-intercorrelated-factor model were adequate (CFI = 0.965; GFI = 0.935; NFI = 0.956; TLI = 0.957; RMSEA = 0.060 (90% CI: 0.055–0.064); SRMR = 0.040).

Convergent and discriminant validity were evaluated following the Fornell and Larcker [131] criterion. The average variance extracted (AVE) values for the three dimensions of burnout and both dimensions of work–family conflict were over the suggested cutoff of 0.50, which ensured adequate convergent validity. Regarding discriminant validity, we compared the square root of the AVE of each latent variable to its correlation with the other latent variables included in the measurement model. As shown in Table 4, the square root of the AVE of each dimension was greater than its correlation with the other dimensions; hence, discriminant validity was confirmed.

### 3.3. Test of Hypotheses

**Figure 2:**
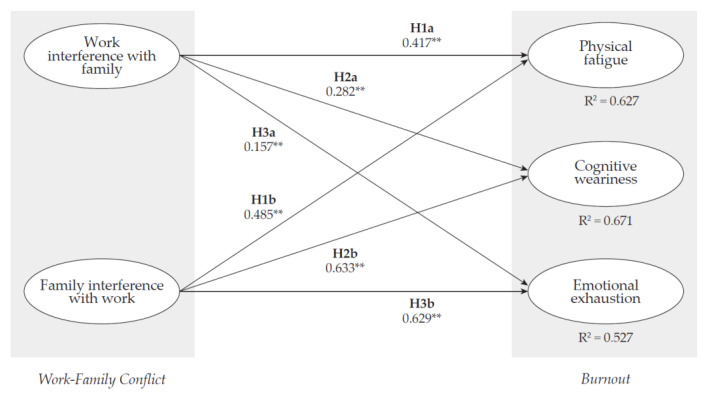
Standardized structural path coefficients. Notes: N = 1044; ** *p* < 0.01.

To evaluate the proposed hypotheses about the influence of work–family conflict on burnout, we estimated a structural equation modeling specification using the overall sample as well as the G_1_ and G_2_ subsamples. For the overall sample, the results from these analyses revealed that the work interference with family dimension has a positive and statistically significant association with physical fatigue (*β* = 0.417, *p* < 0.01), cognitive weariness (*β* = 0.282, *p* < 0.01), and emotional exhaustion (*β* = 0.157, *p* < 0.01), supporting H1a, H2a, and H3a. In addition, evidence showed that the family interference with work dimension has a positive and statistically significant association with physical fatigue (*β* = 0.485, *p* < 0.01), cognitive weariness (*β* = 0.633, *p* < 0.01), and emotional exhaustion (*β* = 0.629, *p* < 0.01). Hence, we confirmed H1b, H2b, and H3b. The goodness-of-fit indices of this specification were satisfactory (CFI = 0.941; GFI = 0.896; NFI = 0.932; TLI = 0.929; RMSEA = 0.077 (90% CI: 0.073–0.081); SRMR = 0.066). Path coefficients estimates are presented in Table 5 and Figure 2.

**Figure 3:**
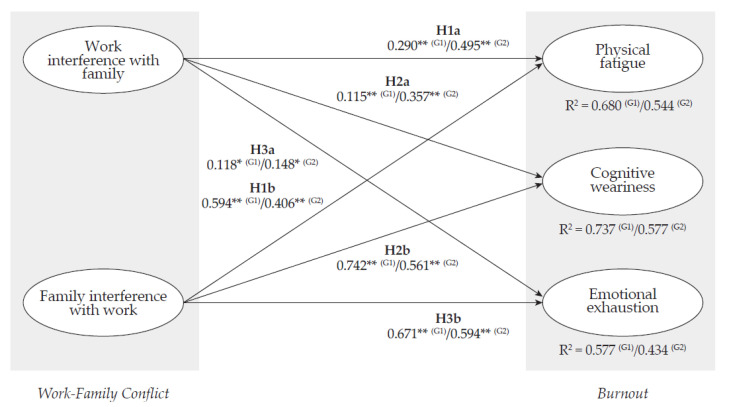
Standardized structural path coefficients for the multigroup analysis. G_1_: teleworked 8 or less hours (*n* = 722); G_2_: teleworked more than 8 h (*n* = 322). * *p* < 0.05; ** *p* < 0.01.

To further understand the effect of teleworking overload, the estimation of the structural equation modeling specification using G_1_ and G_2_ subsamples showed some notable disparities. In both subsamples, the three dimensions of work–family conflict were positive and statistically significant related to both burnout dimensions. When comparing the G_1_ and G_2_ subsamples, we found a stronger relationship in the G_2_ subsample than in the G_1_ subsample between the work interference with family dimension and physical fatigue (G_2_: *β* = 0.495, *p* < 0.01; G_1_: *β* = 0.290, *p* < 0.01), cognitive weariness (G_2_: *β* = 0.357, *p* < 0.01; G_1_: *β* = 0.155, *p* < 0.01), and emotional exhaustion (G_2_: *β* = 0.148, *p* < 0.01; G_1_: *β* = 0.118, *p* < 0.05). On the contrary, we found a greater association in the G_1_ subsample than in the G_2_ subsample between the family interference with work dimension physical fatigue (G_1_: *β* = 0.594, *p* < 0.01; G_2_: *β* = 0.406, *p* < 0.01), cognitive weariness (G_1_: *β* = 0.742, *p* < 0.01; G_2_: *β* = 0.561, *p* < 0.01), and emotional exhaustion (G_1_: *β* = 0.671, *p* < 0.01; G_2_: *β* = 0.594, *p* < 0.01). The goodness-of-fit indices were CFI = 0.946; GFI = 0.899; NFI = 0.933; TLI = 0.935; RMSEA = 0.073 (90% CI: 0.068–0.078); and SRMR = 0.053 in the case of the G1 subsample; and CFI = 0.927; GFI = 0.847; NFI = 0.901; TLI = 0.912; RMSEA = 0.089 (90% CI: 0.081–0.097); and SRMR = 0.092 in the G_2_ subsample. Thus, H1a, H1b, H2a, H2b, H3a, and H3b were supported in both subsamples. Path coefficients estimates for the G_1_ and G_2_ subsamples are presented in Table 6 and Figure 3.

Although some differences arose from comparing the path coefficients between the G_1_ and G_2_ subsample, the multigroup analysis for testing the effect of teleworking overload did not support H4. In Table 7, we report the goodness-of-fit indices for each of the estimated models. The baseline model for testing configural invariance (M_1_) showed an acceptable fit to the data. In addition, these indices were adequate for M_2,_ and the changes in RMSEA and the CFI index were not large enough to reject the factor loadings invariance hypothesis (ΔRMSEA = −0.001; ΔCFI = 0.000). Concerning M_3_ fit indices, RMSEA and CFI did not show variations compared to M_2_, supporting the equal direct effect hypothesis between the G_1_ and G_2_ subsamples. When comparing model M_4_ to M_3_, M_5_ to M_4_, and M_6_ to M_5_, we observed a nonsignificant deterioration of fit, supporting that the teleworking overload did not affect the relationship between the work–family conflict and burnout dimensions.

## 4. Discussion

The three main findings of the study were the following: first, there were high levels of work–family conflict in the surveyed group; second, there was a positive relationship between work–family conflict and family–work conflict and all the dimensions of burnout, and the impact of the work–family conflict manifested primarily in greater exhaustion; third, there was no effect of teleworking overload in the work–family conflict and burnout relationship.

In general, participants reported high scores in both work–family conflict dimensions; however, those who teleworked more than eight hours per day experimented higher levels of work–family conflict. Among other factors, this is explained by the inclusion of greater responsibilities in two vital dimensions, such as work and even school, in the family context on a global level. During the first months of the pandemic, when the data was collected, teleworking was not voluntary and implied a greater intensity of tasks and responsibilities for workers [3]. In addition, for some, teleworking meant saving time in commuting, but on its negative side, this translated into additional time to work, increasing discomfort and conflict between home and work responsibilities [119]. Another factor that should be considered is blurring the boundaries between home and office responsibilities; work emergencies can be omnipresent in the family environment. Finally, to all these concerns due to teleworking was added the anguish about one’s own health, the family wellbeing, the community itself, the global environment, and the uncertainty experienced in a process of continuous change, both in behaviors and habits.

The validation of hypotheses H1a, H1b, H2a, H2b, H3a, and H3b confirmed the negative consequences of teleworking during confinement. There was a positive relationship between the two dimensions of the work–family conflict (work interference with family, and family interference with work) and the three dimensions of burnout (physical fatigue, cognitive weariness, and emotional exhaustion). Hypotheses 1a, 1b, 3a, and 3b results, which showed the impact of work–family conflict on physical fatigue and emotional exhaustion, are consistent with the existing literature [64,140] that supports that both dimensions of work–family conflict are positively related to the emotional exhaustion and cynicism dimensions. The dimensions of cognitive weariness and emotional exhaustion were the most affected as a result of several factors such as the new working conditions during the pandemic [141], the uncertainty, anguish, and anxiety experienced for months due to the fear of infection [59], the new demands of teleworking [2,142], the pressure due to the duality of responsibilities, the operational continuity of businesses, and the new modality of relationships within work teams now mediated by technology [143]. In the case of hypotheses 2a and 2b, the impact on cognitive weariness coincides with the literature on factors such as intensification [122], and it is also explained by factors such as the process of acquiring new work patterns, adaptation to digital processes, and uncertainty about the personal and work future during the pandemic.

Regarding the positive relationship between the work–family conflict dimensions and burnout, some theoretical frameworks such as the job demands–resources model [33] and the role conflict model [95] explain the study results. First, there was an increase in job demands with the teleworking modality. Among those demands, the intensification of family emotional responsibilities, the responsibilities to avoid infection and stay healthy, the economic-related responsibilities, the emerging changes that businesses had to implement to maintain their operations, and the new skills (especially digital skills and remote team working skills) that had to be acquired rapidly stand out. Second, a role conflict was generated due to the loss of the boundaries between the family and work spheres. This meddling of the work dynamics was translated into a dilemma evidenced in this study [123,124].

Concerning hypothesis 4, the non-effect of teleworking overload in the relationship between work–family conflict and burnout during the COVID-19 pandemic contrasted with previous studies [75,144]. This non-effect may be due to contextual factors since, in Ecuador, there was a contingent and regulatory framework for companies that arose during the pandemic. The government regulation that allowed the reduction of working hours and salaries due to pandemic reasons may have impacted the psychological normalization of work overload as a distinguishing factor of teleworking, together with the need for job stability and searching for continuous commitment [145].

A multiplicity of factors could explain the finding of the greatest impact of the work–family conflict on burnout, three of which stand out specifically: role ambiguity [124], the non-willfulness of taking work home during the COVID-19 pandemic, and the loss of limits between home and work [120].

## 5. Conclusions

The consequences on occupational health and the increase of psychosocial risks due to teleworking during the pandemic are continuously growing. On the one hand, the increase in job demands and work intensification manifests itself in a context saturated by two spheres (family and work) that coexist in the same physical environment for workers working from home due to the COVID-19 pandemic. This reality translates into high levels of work–family conflict and burnout. In addition, role conflict is experienced, job demands increase, and job and personal resources to deal with those demands are put to the test. On the other hand, the continuation of the pandemic, non-voluntariness, and a context of economic crisis intensifies the decision to work longer hours, causing a panorama of contradictions in which the worker suffers the illusion of being at home but with the weight of the increase in job responsibilities. According to the study results, this scenario translates into a direct relationship between both dimensions of the work–family conflict and the three dimensions of burnout. It is necessary to deepen the advantages of teleworking, such as spending more time with the family, saving time when commuting, and autonomy.

In work experience, it is essential to analyze the impact of working conditions, social climate, assessment of colleagues and supervisors, and essential aspects as the feeling of a satisfactory experience with the responses to job demands. Overwork generates experiences of dissatisfaction or discomfort that translate into the sense of lack of time, not completing everything adequately, and permanent urgency, among others [146]. Work overload is a psychosocial risk that, despite not having a significant effect on the relationship explored in this study, has an essential role in occupational health, especially in the context of the COVID-19 pandemic.

### 5.1. Limitations and Strengths

The study has some limitations and strengths. Regarding its limitations, the design was cross-sectional, self-reported scales were used to measure the variables of interest, and the sample was incidental. In addition, one of the most important limitations is how work overload was measured since the working hours were used as an indicator of this variable. It could cause distortions in measuring work overload, especially in a multiprofessional sample such as the one used in this study. Although the length of the working day could be considered an indicator of overload, teleworking brings the combination of family and work spheres and flexibility schemes to fulfil work activities, which would imply a challenge for measuring work overload. Further research should include other metrics to measure work overload in the context of teleworking, especially during the COVID-19 pandemic.

Among its strengths, what stands out, to our best knowledge, is that this study is one of the few that addresses the psychosocial consequences of a new modality of work overload and teleworking during the COVID-19 pandemic. Second, the study highlights the importance of the economic and regulatory conditions and contingencies that have surrounded the modality of teleworking during the pandemic in Ecuador, which may have impacted consequences such as the normalization of a factor of high occupational risk, such as work overload. Third, the use of an extensive multiprofessional sample, and fourth, the psychometric validation of the scales used to collect the data.

### 5.2. Implications for Theory and Practice

The problems arising from the adoption of teleworking due to the COVID-19 pandemic allow discussion of the theoretical constructs that underlie this new reality of work. In addition, their adjustment to working modalities as emergency teleworking and psychosocial risks as the overload derived from the pandemic should be included in this discussion. In this study, work overload was measured through working hours, which produced limitations when showing its effect on the relationship between work–family conflict and burnout. However, we believe this is a new era for work design [121] since teleworking in confinement, teleworking itself, social distancing, and hybrid work models are part of our new reality. Much of the theoretical framework and empirical findings are pre-COVID-19, so it is necessary to generate new analyses that reflect people’s and organizations’ realities in contexts such as the COVID-19 pandemic.

From a practical approach, the results of studies such as this are helpful as a starting point for designing policies at various levels. From an organizational perspective, our results should be considered as input for designing psychosocial risks prevention programs and diagnostics on the impact of new work modalities on workers’ health. Regarding public policies, several Latin American countries have incorporated teleworking into their legislation [5,147]; however, this is not enough for its proper implementation. It must be guaranteed that all workers are informed of their health and safety rights and responsibilities, as indicated by the ILO [3]. In particular, there is a gap in public policies regarding supporting workers with minor children, single-parent families, and immigrants, who face greater limitations when entering the labor market and adopting teleworking as a primary working modality.

## Figures and Tables

**Table 1:** Mean and standard deviation for burnout and work–family conflict dimensions.

Dimensions	Overall Sample	Subsamples		
		G_1_	G_2_	*p*-Value (a)
Burnout				
1. Physical fatigue	2.39 (0.90)	2.37 (0.89)	2.45 (0.93)	0.210
2. Cognitive weariness	2.19 (0.91)	2.19 (0.90)	2.18 (0.93)	0.806
3. Emotional exhaustion	2.23 (0.91)	2.26 (0.90)	2.17 (0.93)	0.122
Work–Family Conflict				
4. Work interference with family	2.85 (0.95)	2.73 (0.89)	3.13 (1.02)	< 0.01
5. Family interference with work	2.34 (0.89)	2.35 (0.87)	2.31 (0.94)	0.434

The standard deviations are reported in parentheses. (a): Corresponding p-value of the two-sample *t*-test for mean comparison.

**Table 2:** Standardized factor loadings from confirmatory factor analyses for burnout and work–family conflict.

Items		Overall Sample	Subsamples	
			G_1_	G_2_
Burnout				
Physical fatigue				
bo02	I have no energy for going to work in the morning.	0.764	0.771	0.754
bo03	I feel physically drained.	0.665	0.678	0.650
bo04	I feel fed up.	0.827	0.823	0.834
bo05	I feel like my “batteries” are “dead”.	0.919	0.913	0.925
bo06	I feel burned out.	0.840	0.856	0.832
Cognitive weariness				
bo07	My thinking process is slow.	0.872	0.867	0.883
bo08	I have difficulty concentrating.	0.866	0.866	0.869
bo09	I feel I am not thinking clearly.	0.896	0.887	0.917
bo10	I feel I am not focused on my thinking.	0.916	0.919	0.915
bo11	I have difficulty thinking about complex things.	0.819	0.810	0.840
Emotional exhaustion				
bo12	I feel I am unable to be sensitive to the needs of coworkers and customers.	0.864	0.862	0.855
bo13	I feel I am not capable of investing emotionally in coworkers and customers.	0.851	0.841	0.882
bo14	I feel I am not capable of being sympathetic to coworkers and customers.	0.903	0.871	0.971
Work–Family Conflict				
Work interference with family				
wfc01	After work, I come home too tired to do some of the things I’d like to do.	0.747	0.713	0.795
wfc02	On the job, I have so much work to do that it takes away from my personal interests.	0.884	0.872	0.895
wfc03	My family/friends dislike how often I am preoccupied with my work while I am at home.	0.759	0.735	0.778
wfc04	My work takes up time that I’d like to spend with family/friends.	0.731	0.672	0.818
Family interference with work				
wfc05	I’m often too tired at work because of the things I have to do at home.	0.789	0.803	0.761
wfc06	My personal demands are so great that it takes away from my work.	0.872	0.857	0.896
wfc07	My superiors and peers dislike how often I am preoccupied with my personal life while at work.	0.740	0.725	0.777
wfc08	My personal life takes up time that I’d like to spend at work.	0.719	0.708	0.758

Item bo01 “I feel tired” was dropped from the confirmatory factor analyses for burnout since it presented a factor loading of 0.487 in the overall sample and 0.491 y 0.482 in the G_1_ and G_2_ subsamples, respectively, which did not meet the cutoff suggested by Hair et al. (2019). All factor loadings were statistically significant at a *p* < 0.01 level.

**Table 3:** McDonald’s omega (ω) coefficient estimates for burnout and work–family conflict dimensions.

Dimensions	Overall Sample	Subsamples	
		G_1_	G_2_
Burnout			
1. Physical fatigue	0.882 (95% CI: 0.861–0.897)	0.885 (95% CI: 0.861–0.904)	0.880 (95% CI: 0.857–0.897)
2. Cognitive weariness	0.945 (95% CI: 0.933–0.953)	0.943 (95% CI: 0.931–0.953)	0.949 (95% CI: 0.937–0.957)
3. Emotional exhaustion	0.939 (95% CI: 0.916–0.958)	0.925 (95% CI: 0.899–0.953)	0.964 (95% CI: 0.939–0.988)
Work–Family Conflict			
4. Work interference with family	0.861 (95% CI: 0.845–0.876)	0.835 (95% CI: 0.810–0.857)	0.893 (95% CI: 0.867–0.914)
5. Family interference with work	0.839 (95% CI: 0.812–0.863)	0.830 (95% CI: 0.794–0.861)	0.859 (95% CI: 0.817–0.895)

Note: 95% bias-corrected confidence intervals are shown in parentheses and were computed performing bootstrapping using 2500 bootstrap samples.

**Table 4:** Convergent and discriminant validity analyses.

Dimensions	AVE	Correlations				
		1.	2.	3.	4.	5.
Burnout						
1. Physical fatigue	0.657	0.811				
2. Cognitive weariness	0.764	0.791	0.874			
3. Emotional exhaustion	0.762	0.605	0.735	0.873		
Work–Family Conflict						
4. Work interference with family	0.612	0.655	0.594	0.471	0.782	
5. Family interference with work	0.637	0.653	0.704	0.637	0.586	0.798

AVE: Average variance extracted. The square root of the AVE of each dimension is shown on the diagonal. All correlations were statistically significant at a *p* < 0.01 level.

**Table 5:** Standardized path coefficients for the structural model (overall sample).

Path	Standardized Coefficient	*t*-Value	Hypothesis
Work interference with family → Physical fatigue	0.417	12.310 **	H1a supported
Work interference with family → Cognitive weariness	0.282	9.531 **	H2a supported
Work interference with family → Emotional exhaustion	0.157	4.845 **	H3a supported
Family interference with work → Physical fatigue	0.485	13.996 **	H1b supported
Family interference with work → Cognitive weariness	0.633	18.662 **	H2b supported
Family interference with work → Emotional exhaustion	0.629	16.734 **	H3b supported

** *p* < 0.01.

**Table 6:** Standardized path coefficients for structural model (G_1_ and G_2_ subsamples).

	Standardized Coefficient	*t*-Value	Hypothesis
G_1_: Teleworked 8 or less hours			
Work interference with family → Physical fatigue	0.290	6.075 **	H1a supported
Work interference with family → Cognitive weariness	0.115	3.517 **	H2a supported
Work interference with family → Emotional exhaustion	0.118	2.330 *	H3a supported
Family interference with work → Physical fatigue	0.594	11.472 **	H1b supported
Family interference with work → Cognitive weariness	0.742	14.517 **	H2b supported
Family interference with work → Emotional exhaustion	0.671	11.950 **	H3b supported
G_2_: Teleworked more than 8 h			
Work interference with family → Physical fatigue	0.495	8.504 **	H1a supported
Work interference with family → Cognitive weariness	0.357	7.208 **	H2a supported
Work interference with family → Emotional exhaustion	0.148	3.094 **	H3a supported
Family interference with work → Physical fatigue	0.406	7.286 **	H1b supported
Family interference with work → Cognitive weariness	0.561	10.329 **	H2b supported
Family interference with work → Emotional exhaustion	0.594	10.199 **	H3b supported

* *p* < 0.05; ** *p* < 0.01.

**Table 7:** Multigroup analyses results for teleworking overload effects.

Models	TLI	CFI	RMSEA (90% CI)	ΔCFI	ΔRMSEA
M_1_. Unconstrained	0.927	0.939	0.055 (0.052–0.058)		
M_2_. Equal factor loadings	0.930	0.939	0.054 (0.051–0.057)	0.000	−0.001
M_3_. Equal direct effects	0.931	0.939	0.054 (0.051–0.057)	0.000	0.000
M_4_. Equal structural variances/covariances	0.927	0.935	0.055 (0.052–0.058)	−0.004	0.001
M_5_. Equal structural residual variances/covariances	0.927	0.934	0.055 (0.053–0.058)	−0.001	0.000
M_6_. Equal measurement error variances/covariances	0.928	0.930	0.055 (0.052–0.058)	−0.004	0.000

TLI = Tucker–Lewis fit index; CFI = comparative fit index; RMSEA = root mean square of approximation.

## Data Availability

The data presented in this study are available on request to the corresponding authors. The data are not publicly available due to privacy concerns.
